# Human CKAP2L shows a cell cycle‐dependent expression pattern and exhibits microtubule‐stabilizing properties

**DOI:** 10.1002/2211-5463.13864

**Published:** 2024-07-28

**Authors:** Hyerim Kwon, Jonathan Y. Joh, Kyung U. Hong

**Affiliations:** ^1^ School of Medicine Sungkyunkwan University Suwon Korea; ^2^ Department of Pharmacology & Toxicology University of Louisville School of Medicine KY USA; ^3^ College of Pharmacy and Health Sciences Western New England University Springfield MA USA

**Keywords:** cell cycle, cytoskeleton‐associated protein 2, cytoskeleton‐associated protein 2‐like, microtubule, mitosis, mitotic spindle

## Abstract

Cytoskeleton‐associated protein 2‐like (CKAP2L) is a paralogue of cytoskeleton‐associated protein 2 (CKAP2). We characterized the expression pattern, subcellular localization, and microtubule‐stabilizing properties of human CKAP2L. The levels of both CKAP2L transcript and protein were cell cycle phase‐dependent, peaking during the G_2_/M phase and relatively high in certain human tissues, including testis, intestine, and spleen. CKAP2L protein was detectable in all human cancer cell lines we tested. CKAP2L localized to the mitotic spindle apparatus during mitosis, as reported previously. During interphase, however, CKAP2L localized mainly to the nucleus. Ectopic overexpression of CKAP2L resulted in ‘microtubule bundling’, and, consequently, an elevated CKAP2L level led to prolonged mitosis. These findings support the mitotic role of CKAP2L during the human cell cycle.

## Graphical abstract

CKAP2L (cytoskeleton‐associated protein 2‐like) is a relatively new mitotic spindle‐associated protein, associated with neural progenitors as well as various cancers. Its basic characteristics, however, are poorly defined. Our study shows (1) the expression pattern of human CKAP2L in different cell cycle phases, tissues, and cancer cell lines; (2) cell cycle‐dependent subcellular localization; (3) microtubule‐stabilizing property of CKAP2L.

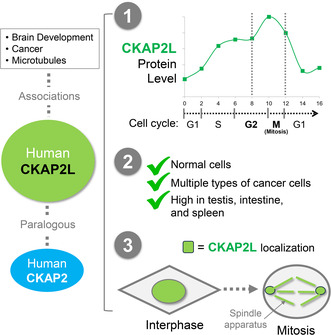

**Abbreviations**
APC/C: anaphase promoting complex/cyclosome
CKAP2: cytoskeleton‐associated protein 2
CKAP2L: cytoskeleton‐associated protein 2‐like
GFP: green fluorescent protein
GST: glutathione *S*‐transferase
HRP: horse radish peroxidase
PBS: phosphate‐buffered saline
pHH3: phosphorylated histone H3
PVDF: polyvinylidene difluoride
RT: room temperature
RT‐PCR: reverse transcriptase‐polymerase chain reaction
SDS: sodium dodecyl sulfate
TBST: tris‐buffered saline containing Tween 20

Regulation of the assembly and dynamics of the bipolar mitotic spindle ensures for faithful segregation of chromosomes at the end of the mammalian cell cycle. During mammalian neurogenesis, proliferation and cell division of neural stem cells give rise to neurons and glial cells [1]. Mutations in genes encoding for centrosome or spindle components have been shown to cause microcephaly [2, 3, 4], suggesting that proper bipolar mitotic spindle assembly is crucial for neurogenesis. In 2014, it was reported that mutations in a previously unknown gene, cytoskeleton‐associated protein 2‐like (*CKAP2L*), cause Filippi syndrome, a rare genetic disorder characterized by microcephaly and mental retardation [5].

CKAP2L, also known as FLJ40629 and radial fiber and mitotic spindle (Radmis), is the only known paralogue of cytoskeleton‐associated protein 2 (CKAP2), also known as tumor‐associated microtubule‐associated protein (TMAP), LB1, and CTCL tumor antigen se20‐10. Previously, we and others reported that CKAP2 is a regulator of mitotic spindle assembly and essential for proper chromosome segregation [6, 7, 8, 9, 10, 11], and serves as a useful prognostic marker in human cancer [12, 13]. Similarly, CKAP2L was first reported as a novel mitotic spindle protein in 2013. Yumoto *et al*. [14] discovered that expression of murine *Ckap2l* is enriched in regions of active neurogenesis both prenatally and postnatally, and it localizes to mitotic spindles as well as radial fibers of proliferating neural stem/progenitor cells. The authors also reported that overexpression of *Ckap2l* in HEK293 cells and a neuroblastoma cell line results in excessive stabilization of microtubules and aberrant mitotic spindle formation. Consistent with this finding, overexpression of *Ckap2l* in utero leads to defects such as a reduction in proliferation and a concomitant increase in cell cycle exit among neural stem/progenitor cells. A loss‐of‐function study using *Ckap2l*‐specific shRNAs in NIH3T3/13C7 cells showed that depletion of CKAP2L results in severe disorganization of the mitotic spindles and chromosome separation [14]. These findings demonstrated that, similar to its paralogue, CKAP2L is a mitotic spindle protein, essential for faithful chromosome segregation in dividing cells.

More recently, studies have identified CKAP2L as a prognostic marker of various human cancer, including esophageal squamous cell carcinoma, hepatocellular carcinoma, lung adenocarcinoma, and clear cell renal carcinoma [15, 16, 17, 18]. Knockdown of CKAP2L in various cancer cell lines has been shown to result in a G_2_/M arrest and retardation of the growth and migratory potential of the cancer cells [16, 19, 20, 21], highlighting the potential role of CKAP2L in cancer cell growth and metastasis.

In order to further enhance our understanding of CKAP2L biology and its role during the mammalian cell cycle, we (a) characterized the cell cycle‐dependent and tissue‐specific expression of CKAP2L; (b) cell cycle phase‐dependent (e.g., interphase vs. mitosis) changes in its subcellular localization; and (c) confirmed its microtubule‐stabilizing properties in the present study.

## Materials and methods

### Cell culture

Human foreskin fibroblasts (Cat. No. SCRC‐1041; RRID:CVCL_XB54) and HEK293 (Cat. No. CRL‐1573; RRID:CVCL_0045) cells were purchased from ATCC and cultured in DMEM containing 10% fetal bovine serum and antibiotics and were subcultured every 3 days. All cell lines have been authenticated in the past 3 years because all experiments were performed within 3 years of their purchase from ATCC (Manassas, VA, USA). All experiments were performed with mycoplasma‐free cells.

### Cell synchronization aphidicolin and release

Subconfluent human foreskin fibroblasts were pre‐synchronized by serum starvation for 1 day. The cells were then stimulated to reenter the cell cycle by placing them in a complete media containing 4 μg·mL^−1^ aphidicolin (Sigma‐Aldrich; St. Louis, MO, USA) for 14 h. The cells were released from the G_1_‐S arrest by rinsing in phosphate‐buffered saline (PBS) and replenishing complete media without aphidicolin. Cells were harvested at 0–16 h post‐release and analyzed by RT‐PCR, western blot, and flow cytometry (see other sections).

### Reverse transcriptase‐polymerase chain reaction (RT‐PCR)

A set of total RNA isolated from the indicated human tissues (Human Total RNA Master Panel II) and mouse tissues was purchased from Clontech/Takara (San Jose, CA, USA). Total RNA was isolated from the cells using TRIzol® reagent (Invitrogen; Waltham, MA, USA), according to the manufacturer's instructions. RNA was then converted to cDNA using High‐Capacity cDNA Reverse Transcription Kit (Thermo Fisher; Waltham, MA, USA). One microliter of cDNA was used to amplify the gene of interest in a 20‐μL Taq polymerase (Perkin‐Elmer; Waltham, MA, USA) PCR. The primer sequences were the following: human *CKAP2L* (forward 5′‐CAGTGGAAGAGCTGGCC‐3′ and reverse 5′‐TTATGATTCAGGGGTTTG‐3′); mouse *Ckap2l* (forward 5′‐AGAAATGGAGCCTATTGCAG‐3′ and reverse 5′‐TCATGATTCAAGGACTTC‐3′); human *CKAP2* (forward 5′‐ATTGAAGAGATGCGACACAC‐3′ and reverse 5′‐TTATGTTGTATC AGCCTCATA‐3′); mouse *Ckap2* (forward 5′‐GCGCAGATACACGATTGCAG‐3′ and reverse 5′‐GAGGAGGTCGTTTTAGCCCC‐3′); human *CCNB1* (forward 5′‐AGGTTGTTGCAGGAGACCATGT‐3′ and reverse 5′‐AGGTGCTGCATAACTGGAAGAA‐3′); human *TOP2A* (forward 5′‐CAAGCCCTCCTGCTACACAT‐3′ and reverse 5′‐GTGGATGGCTTCCTTTTGCG‐3′); human *GAPDH* (forward 5′‐CTTCAACAGCGACACCCACTCCTC‐3′ and reverse 5′‐GGCCCCTCCCCTCTTCAA‐3′); mouse *Gapdh* (forward 5′‐ACCCCAGCAAGGACACTGAGCAAG‐3′ and reverse 5′‐TGGGGGTCTGGGATGGAAATTGTG‐3′). For semi‐quantitative RT‐PCR, samples were run for 20–27 PCR cycles (95 °C for 30 s; 60 °C for 30 s; and 72 °C for 30 s) depending on the gene of interest. The resulting PCR products were separated on 1.2% agarose gels containing 5 ng·mL^−1^ ethidium bromide and visualized under UV light.

### Western blot analysis

Transfected or treated cells were harvested in Laemmli buffer (50 mm Tris‐Cl [pH 6.8], 2% sodium dodecyl sulfate [SDS; wt/vol], 0.1% bromophenol blue, 10% [vol/vol] glycerol). The samples were heated in boiling water for 10 min. After the protein concentration was determined by using a bicinchoninic acid protein assay, β‐mercaptoethanol was added to each sample (1 μL per 100 μL lysate) which was then heated in boiling water for another 5 min. Protein samples (25 or 50 μg) were resolved by SDS/polyacrylamide gel electrophoresis (PAGE) and transferred to a polyvinylidene difluoride (PVDF) membrane (Millipore; Burlington, MA, USA). Following 30 min of incubation in the blocking solution (5% skim milk in tris‐buffered saline‐Tween® 20; TBST) at room temperature, the blot was incubated with an appropriate dilution of the primary antibody (in the blocking solution) for 1 h at room temperature or overnight at 4 °C. The blot was washed twice in TBST and incubated with an appropriate HRP‐conjugated secondary antibody (diluted in the blocking solution) for 1 h at room temperature. The antibody–antigen complex was then detected using SuperSignal West Pico solution (Pierce; Waltham, MA, USA). Western blot images were acquired using the LAS‐3000 imaging system (FujiFilm; Valhalla, NY, USA).

### Flow cytometry

Cells were fixed in cold 75% ethanol at the time of harvest and kept at −20 °C overnight or longer. They were spun down, washed in PBS containing 1% bovine serum albumin, and incubated in the propidium iodide (PI) solution containing 50 μg·mL^−1^ PI, 0.1% sodium citrate, 0.3% NP‐40, 50 μg·mL^−1^ RNase A, and 1× PBS for 1 h at 37 °C. DNA profiles of PI‐stained cells were obtained using a FACSCalibur system (Becton Dickinson; Franklin Lakes, NJ, USA) and analyzed using cellquest software, version 3.3 (Becton Dickinson; Franklin Lakes, NJ, USA).

### Antibodies

For the generation of rabbit polyclonal antiserum against human CKAP2L, glutathione *S*‐transferase (GST)‐CKAP2L (1–458 aa) was expressed in bacteria and purified using glutathione‐Sepharose beads and eluted by thrombin digestion as previously described [22]. Rabbits were then immunized once a week with purified CKAP2L mixed with Freund's adjuvant (Sigma) for 4–6 weeks before the antiserum was obtained. CKAP2L‐specific antibodies were then affinity‐purified as previously described [9]. The specificity of rabbit polyclonal anti‐CKAP2L antibody is shown in Figs S1 and S2. Mouse monoclonal antibody against human CKAP2 was produced as previously described [23]. Mouse monoclonal antibodies against α‐tubulin (clone B‐5‐1‐2) and cyclin B1 (clone GNS1) were purchased from Sigma and Santa Cruz (Dallas, TX, USA), respectively. Rabbit polyclonal antibody against phospho‐Histone H3 (Ser10) was purchased from Upstate. AlexaFluor 488‐conjugated anti‐rabbit IgG and Cy3‐conjugated anti‐mouse IgG were purchased from Molecular Probes (Eugene, OR, USA) and Rockland (Royersford, PA, USA), respectively. Generation and characterization of rabbit polyclonal antiserum to human CKAP2 have been previously described [24].

### Immunofluorescence staining and image acquisition

HEK293 cells were plated on glass coverslips. After the indicated treatment (e.g., transfection), the cells were fixed in 3.7% formaldehyde in PBS for 15 min at room temperature (RT), followed by permeabilization in 0.5% Triton X‐100 in PBS for 10 min at RT. Blocking was done in the blocking solution (5% bovine serum albumin in PBS) for 30 min; the cells were incubated in primary antibody solution (diluted in the blocking solution) for 1 h at RT; and washed twice in PBS. They were then incubated with secondary antibodies conjugated to fluorochromes (diluted in the blocking solution) for 1 h at room temperature and washed twice in PBS. They were finally counterstained with DAPI (4′,6′‐diamidino‐2‐phenylindole) (Sigma‐Aldrich; St. Louis, MO, USA) and mounted on glass slides using Gel/Mount (Biomeda; San Jose, CA, USA). Primary antibody dilutions used for the immunofluorescence staining were the following: 1 : 300 dilution for CKAP2L and 1 : 5000 for α‐tubulin. All fluorochrome‐conjugated secondary antibodies were used at 1 : 200 dilution. Fluorescence images were viewed and acquired using an Axioplan 2 microscope (Carl Zeiss; White Plains, NY, USA) equipped with an AxioCam charge‐coupled device camera (Carl Zeiss; White Plains, NY, USA). The overlaying and merging of images were done using Adobe Photoshop 7.0 (Adobe; San Jose, CA, USA).

### GFP‐fusion human CKAP2L expression construct and transient transfection

The open reading frame of human CKAP2L was PCR amplified using PfuUltra (Agilent; Santa Clara, CA, USA) from HeLa cell cDNA using the following primers: FLJ40629 forward, 5′‐AGAATTCATGGTGGGGCCCGG‐3′ (EcoRI site underlined) and FLJ40629 reverse, 5′‐AAGTCGACTTATGATTCAGGGGTTTG‐3′ (SalI site underlined). The resulting 2.2‐kb PCR product was column‐purified; double‐digested with EcoRI and SalI; subcloned into EcoRI‐SalI sites of pEGFP‐C2 (Clontech); and sequence verified. The cloned product was identical to the coding sequence of NM_152515, homo sapiens cytoskeleton‐associated protein 2 like (CKAP2L), transcript variant 1, mRNA. For transfection of plasmid DNA, Lipofectamine Plus (Invitrogen) reagent was used according to the manufacturer's instructions.

### Time‐lapse video microscopy

HEK293 cells on a 6‐well plate were transfected with a GFP‐CKAP2L expression plasmid. One day after transfection, 2‐channel time‐lapse video microscopy was performed using a fully motorized Axiovert 200M microscope (Carl Zeiss), equipped with Axiocam HRm. Temperature and CO_2_ control were maintained using the Incubator S‐M and Heating insert M06 controlled by Tempcontrol 37‐2 and CTI‐Controller 3700. Both phase contrast and GFP fluorescence images were acquired for 48 h with a lapse time of 5 min using axiovision 4.3 software (Carl Zeiss). Images were acquired using a 20× objective (LD Plan‐Neofluar 20×/0.4 Corr Ph2; Carl Zeiss).

### Nuclear and cytosolic fractions

Asynchronous HEK293 cells were washed in PBS and incubated in lysis buffer (10 mm Tris–HCl, pH 7.8, 1% NP‐40, 10 mm, β‐mercaptoethanol, and protease inhibitor cocktail) at 4 °C for 5 min. An equal volume of deionized water was added and incubated for an additional 5 min. The cells were carefully sheared using a 22‐gauge syringe. The cell lysate was centrifuged at 400 ***g*** for 6 min to separate the pellet (nuclear fraction) from the supernatant (cytosolic fraction). Each fraction was added with an equal volume of 2× Laemmli buffer and processed for western blot as described above.

## Results

### Cell cycle‐dependent expression of CKAP2L

**Fig. 1:**
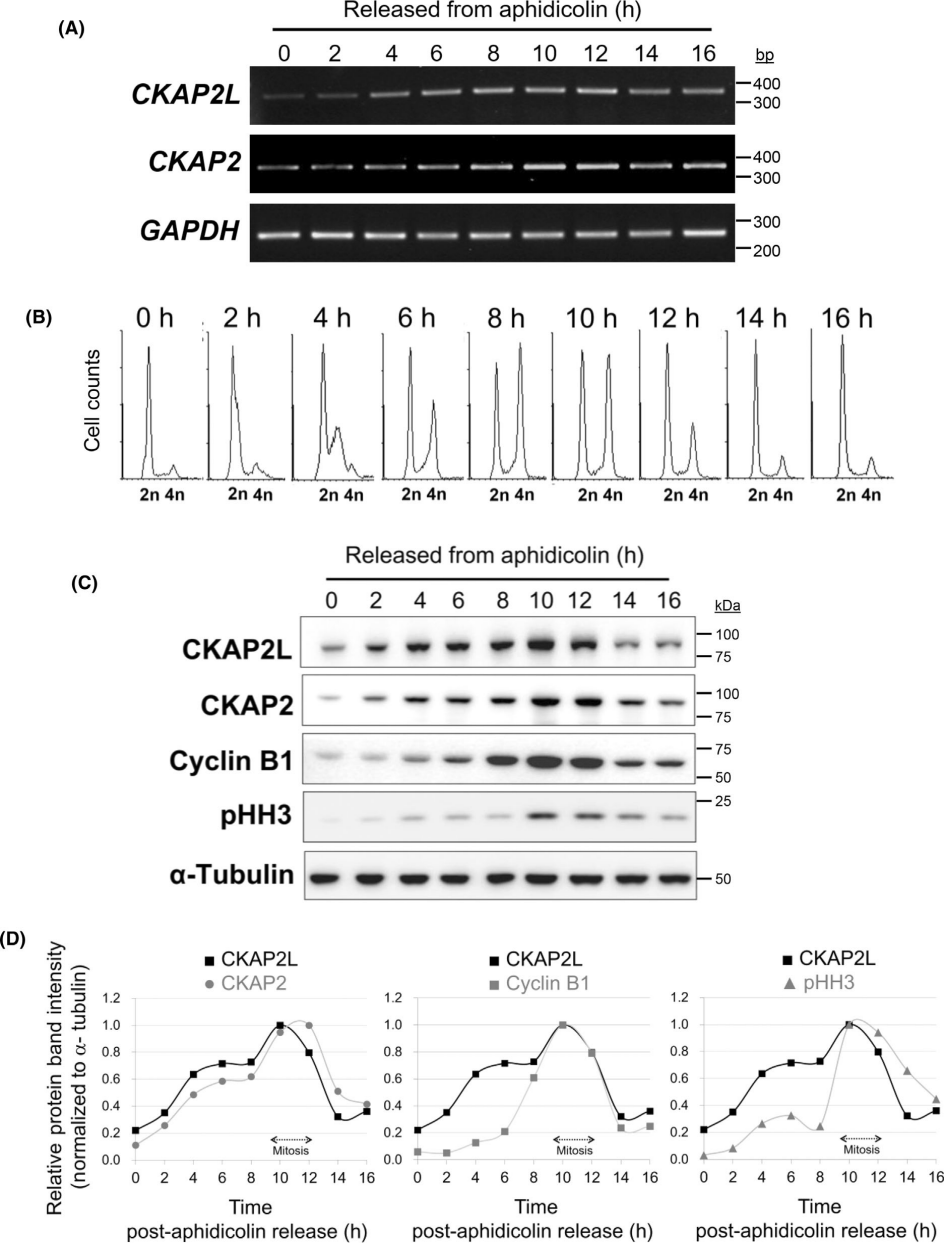
Cell cycle‐dependent changes in CKAP2L transcript and protein levels in human foreskin fibroblasts. Normal human foreskin fibroblasts were arrested at the G_1_‐S boundary using aphidicolin; released; and harvested at the indicated time points. (A) The cells were analyzed by RT‐PCR to determine the relative abundance of human *CKAP2L* mRNA. CKAP2 mRNA was detected as a comparison. *GAPDH* served as a loading control. (B) The cells were analyzed by western blot to determine the relative abundance of human CKAP2L, CKAP2, cyclin B1, and phospho‐histone H3 at Ser10 (pHH3) protein. α‐tubulin served as a loading control. (C) The samples from the indicated time points were analyzed by flow cytometry to monitor the cell cycle progression. See Fig. S5 for the axial values. (D) The time‐ and cell cycle‐dependent changes in CKAP2L protein level and the indicated proteins. the intensities of protein bands shown in Panel B were quantified using Science Lab 2001 Image Gauge Ver. 4.0 and normalized to those of α‐tubulin bands. For each protein, the highest band intensity was set as the reference (i.e., 1.0), and the relative intensities of bands (for each protein) are shown here. For the ease of comparison between the levels of CKAP2L and the indicated protein, the CKAP2L curve was reproduced for each panel.

Recently, Liu *et al*. [18] reported that human *CKAP2L* is co‐expressed with *TOP2A*, *BUB1B*, *MKI67*, and *DLGAP5* in clear cell renal carcinoma. These co‐expressed genes are regulated in a cell cycle‐dependent manner, with their transcript levels peaking at the G_2_/M phases of the cell cycle [25, 26]. Similarly, we have previously reported that the transcript and protein levels of CKAP2, the paralogue of CKAPL, start to rise during G_1_/S and peak at G_2_/M, after which it decreases abruptly [24, 27]. This prompted us to investigate if CKAP2L shows a cell cycle‐dependent expression pattern at the transcript and protein levels. Normal human foreskin fibroblasts were synchronized at G_1_/S using aphidicolin; released; and harvested at a regular interval of up to 16 h. Flow cytometric analysis was performed with the sample from each time point to monitor the progression through the cell cycle. At 0 h (i.e., at the G_1_‐S boundary), the *CKAP2L* transcript level was relatively low. It gradually increased, however, as the cells progressed through the cell cycle and peaked approximately at 10–12 h (Fig. 1A), during which the cells were undergoing the mitotic (M) phase or entering the G_0_/G_1_ phase, according to the flow cytometric analysis (Fig. 1B and Fig. S3). Finally, its transcript level started to decrease, starting at 14–16 h (Fig. 1A), as the cells exit mitosis and enter G_0_/G_1_ (Fig. 1B and Fig. S3). Changes in the mRNA level of its paralogue, *CKAP2*, during the cell cycle closely resembled that of *CKAP2L*, as previously reported [24] (Fig. 1A).

We then assessed the cell cycle‐dependent changes in CKAP2L protein level by western blot. For detection of human CKAP2L protein, rabbit polyclonal antibody against the first 455 amino acids of human CKAP2L (of 745 amino acids; UniProt Q8IYA6‐1) was generated and affinity‐purified (Fig. S1). For comparison, cyclin B1 and phospho‐histone H3 (pHH3) were also analyzed to detect proteins enriched in G_2_ and mitosis, respectively. The CKAP2L protein level was relatively low at 0 h (i.e., at the G_1_‐S boundary), yet gradually increased with the progression of the cell cycle (Fig. 1C). It reached its peak at 10 h (Fig. 1C) during which most cells were undergoing G_2_/M phases (Fig. 1B and Fig. S3). In support of this, cyclin B1 and pHH3 levels also reached the maximum at 10 h (Fig. 1C,D). After 12 h, as the cells exit mitosis and enter the G_0_/G_1_ phase, the protein level of CKAP2L diminished abruptly (Fig. 1B,C), suggesting that the protein was actively degraded after mitosis. The cell cycle‐dependent changes in the protein level of its paralogue, CKAP2, mirrored those of CKAP2L (Fig. 1C,D). These results demonstrated that, like CKAP2, (a) CKAP2L shows cell cycle‐dependent expression pattern, and (b) CKAP2L expression peaks during G_2_/M phases of the cell cycle.

### CKAP2L expression in organs/tissues and cell lines

**Fig. 2:**
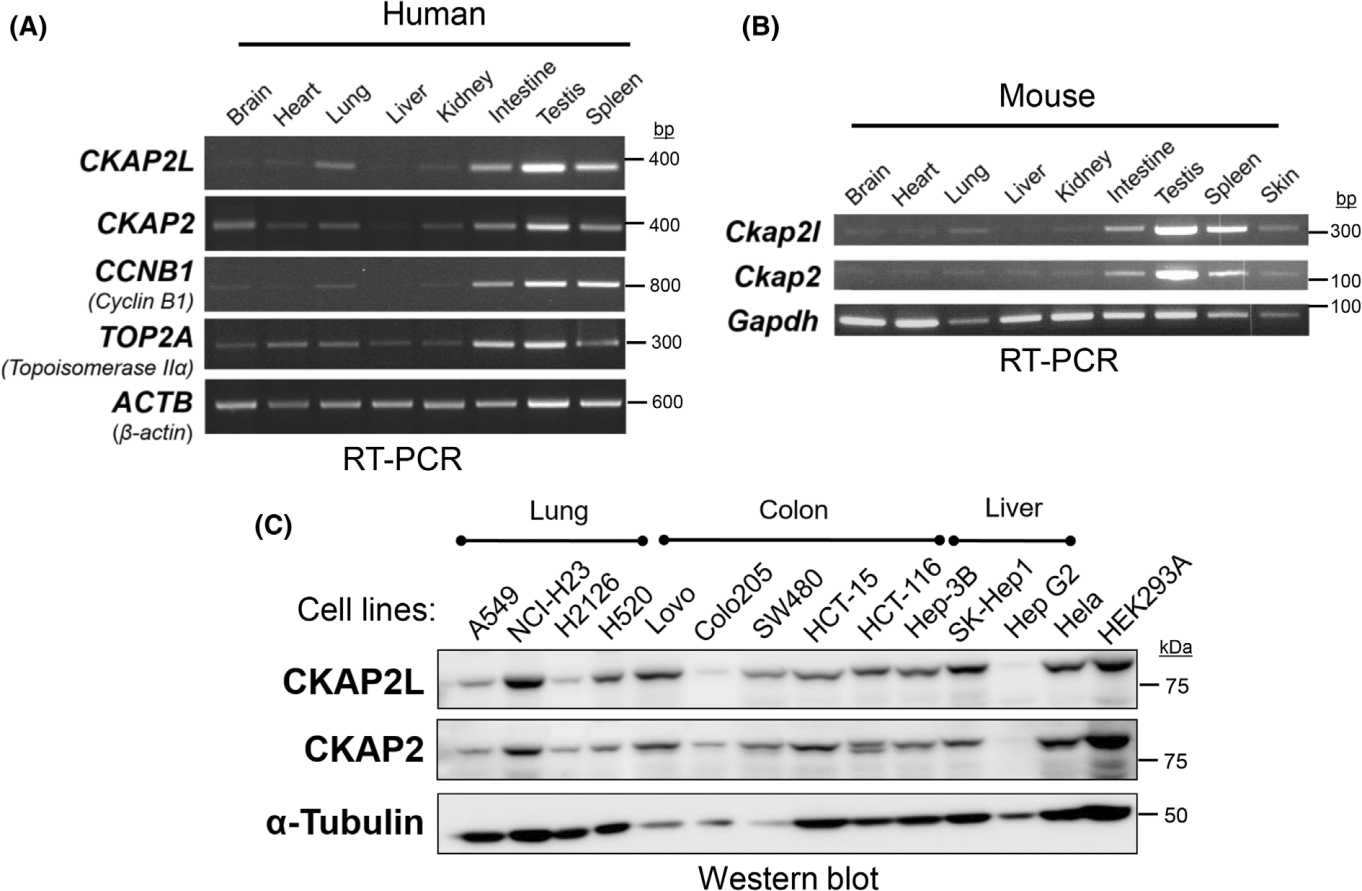
Expression of CKAP2L in human and mouse tissues and human cell lines. (A) The transcript levels of human *CKAP2L*, along with *CKAP2*, *CCNB1*, and *TOP2A*, were measured by RT‐PCR in various human organs and tissues. *ACTB* served as a loading control. (B) The transcript levels of mouse *Ckap2l* and *Ckap2* were measured by RT‐PCR in various mouse organs and tissues. *Gapdh* served as a loading control. (C) The protein levels of human CKAP2L and CKAP2 were measured in the indicated human cell lines of different tissue origins by western blot. α‐tubulin served as a loading control.

We examined the pattern of *CKAP2L* gene expression in various human and mouse tissues by RT‐PCR. For comparison, the mRNA levels of *CKAP2*, cyclin B1 (*CCNB1*), and topoisomerase II alpha (*TOP2A*), all of which have been shown to be enriched in the G_2_/M phase [24, 25], were also measured. In both human and mouse tissues, the mRNA level of human *CKAP2L* (Fig. 2A) or murine *Ckap2l* (Fig. 2B) was the highest in the testis, followed by the spleen and intestine. *CKAP2L* or *Ckap2l* mRNA was also detected in other tissues, such as the lung, kidney, and skin, but the levels were relatively low compared to those found in the testis or spleen (Fig. 2A,B). Other genes enriched at G_2_/M (i.e., *CKAP2*, *CCNB1*, and *TOP2A*) shared a similar tissue‐specific expression pattern, although *CKAP2* and *TOP2A* expression appeared wider spread.

Various human cancer cell lines, including those originating from the lung, colon, and liver, were examined for CKAP2L protein expression using a CKAP2L‐specific antibody (Fig. 2C). For comparison, its paralogue, CKAP2, was examined in the same samples. Although variable, every cancer cell line we tested expressed detectable CKAP2L protein (Fig. 2C), and we did not recognize any noticeable pattern in its expression. Interestingly, the relative differences in CKAP2L and CKAP2 protein levels between cell lines were almost identical, suggesting that the expression of two paralogues is likely to be co‐regulated in cancer cells.

### Subcellular localization of human CKAP2L during mitosis

**Fig. 3:**
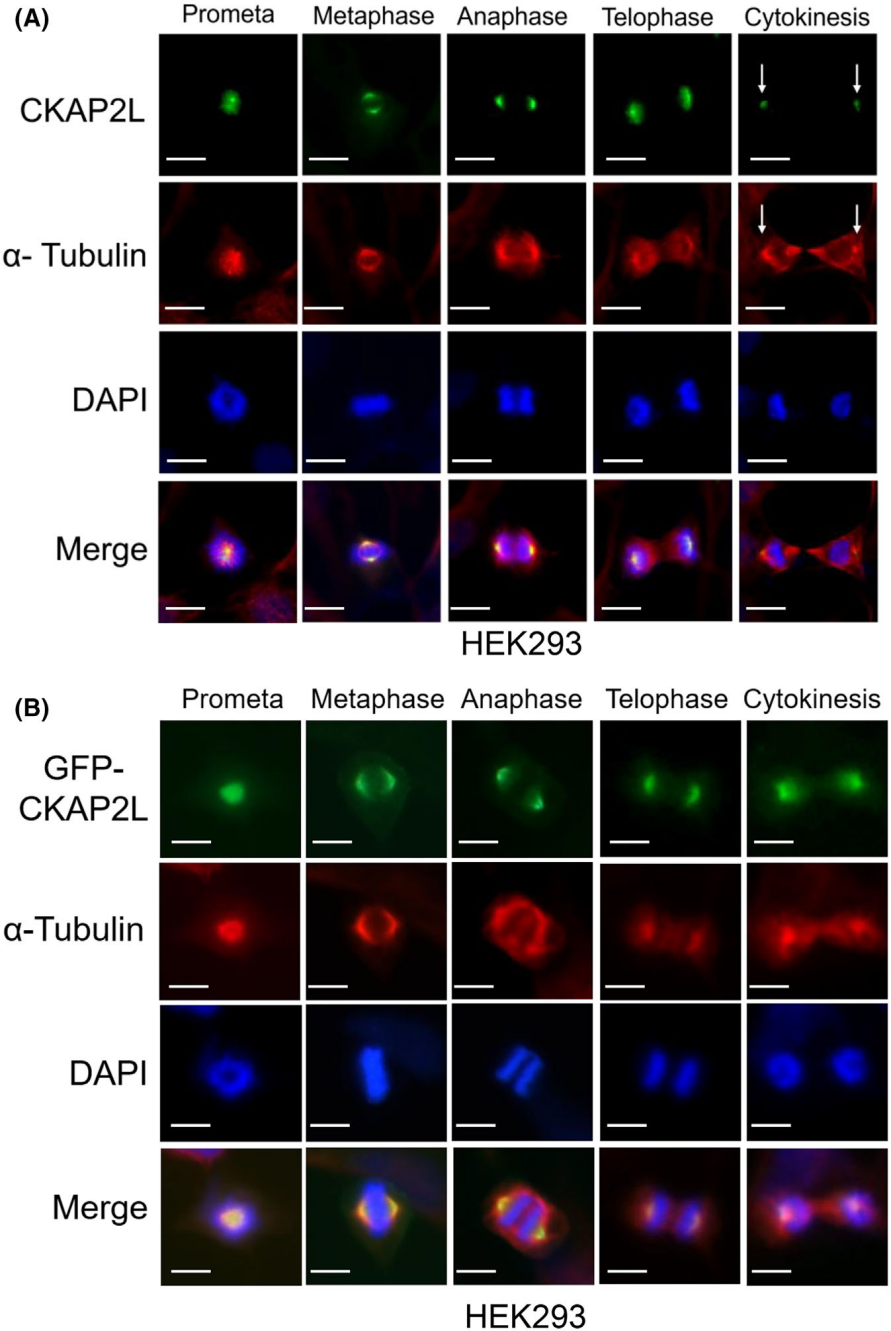
Subcellular localization of endogenous CKAP2L and GFP‐CKAP2L during mitosis. (A) HEK293 cells were co‐immunostained with a rabbit polyclonal anti‐human CKAP2L antibody (Alexa488; green) and a monoclonal antibody against α‐tubulin (Cy3; red). DAPI was used to stain nuclei (blue). Panels show representative images of cells at different phases of mitosis showing subcellular localization of endogenous CKAP2L. In cells undergoing cytokinesis, two foci of CKAP2L staining (arrows) were observed. (B) HEK293 cells were transfected with a GFP‐CKAP2L expression construct. On the following day, the cells were fixed and stained for α‐tubulin (Cy3; red) and nuclei (DAPI; blue). Localization of GFP‐CKAP2L signal during different phases of mitosis is shown in green and resembled that of endogenous CKAP2L. Scale bars 10 μm.

Yumoto *et al*. [14] originally reported on the subcellular localization of mouse Radmis/CKAP2L in mouse neural stem/progenitor cells and N2a mouse neuroblastoma cell line. In both cases, mouse CKAP2L localized to the mitotic spindle during mitosis. Interestingly, mouse CKAP2L was absent in prophase, but it appeared and localized to centrosomes and spindle microtubules, starting at prometaphase. During cytokinesis, it was co‐localized with αγ‐tubulin in the midbody microtubules but absent from the spindle poles [14]. We sought to confirm these findings in cells of human origin. HEK293 cells were fixed and stained with a rabbit polyclonal anti‐human CKAP2L antibody. The cells were co‐stained for α‐tubulin to visualize microtubules. Similar to mouse CKAP2L [14], human CKAP2L localized to the spindle poles and spindle microtubules at prometaphase, metaphase, anaphase, and telophase (Fig. 3A and Fig. S4). Notably, CKAP2L in mitotic cells did not appear to completely overlap with microtubules shown by the α‐tubulin staining. Its localization/staining appeared stronger at the spindle poles and the spindle microtubules close to the poles (Fig. 3A). During cytokinesis, it localized only to the remaining spindle poles (i.e., centrosomes) (see arrows in Fig. 3A and Fig. S4). We did not see prominent CKAP2L staining on the midbody microtubules, in contrast to what Yumoto *et al*. [14] have reported in N2a mouse cell line. In addition, a similar pattern of subcellular localization of CKAP2L was also observed in HeLa cells and human foreskin fibroblasts during mitosis (data not shown). To further corroborate this finding, a GFP‐fusion human CKAP2L expression vector (GFP‐CKAP2L; human CKAP2L fused to the C‐terminus of EGFP) was constructed and introduced into HEK293 cells. The pattern of subcellular localization of GFP‐CKAP2L was identical to that of endogenous CKAP2L (Fig. 3B), which complemented the CKAP2L antibody staining.

### CKAP2L localizes to the nucleus during interphase

**Fig. 4:**
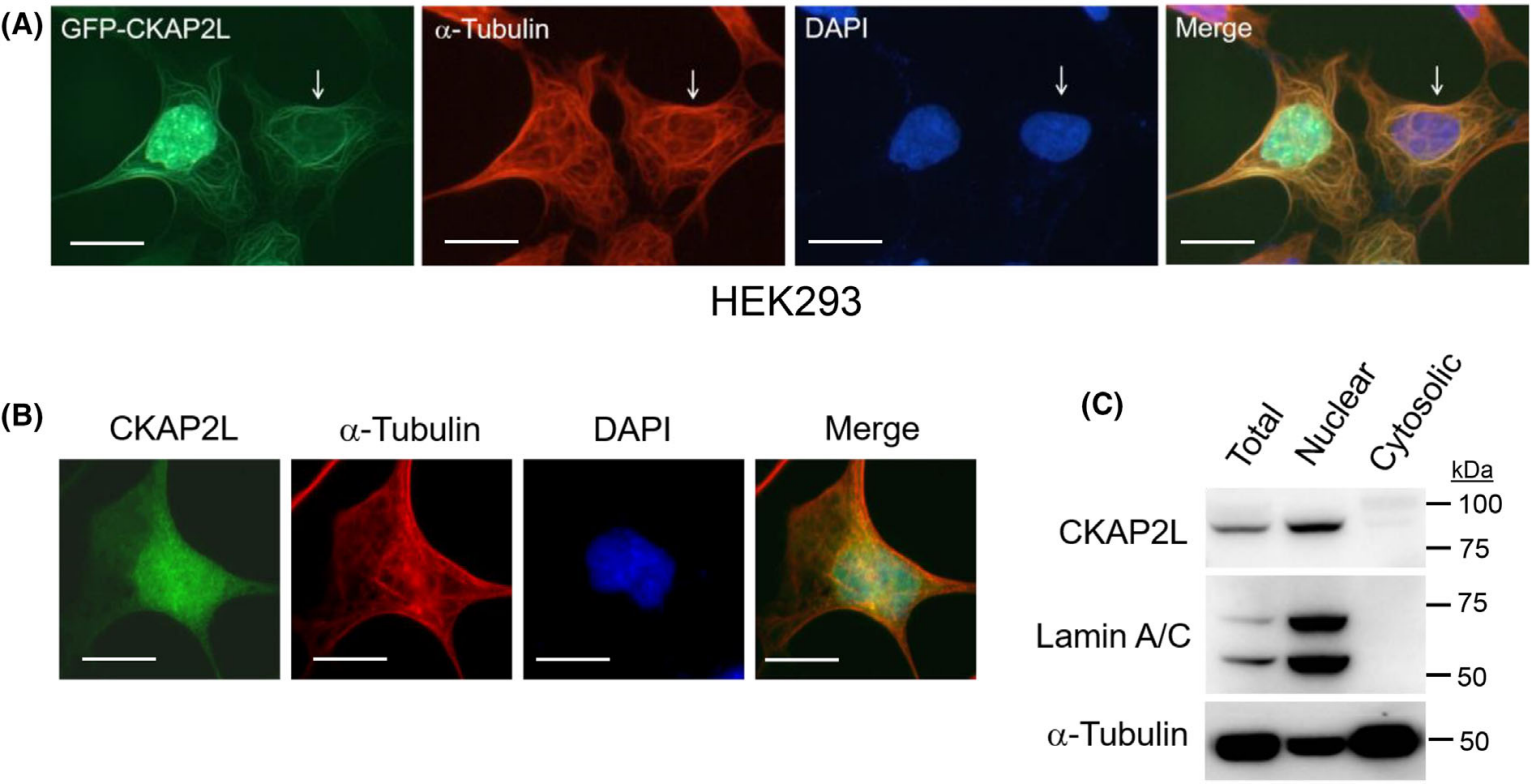
Endogenous CKAP2L localizes to the nucleus during interphase. (A) HEK293 cells were transfected with a GFP‐CKAP2L (green) expression construct; fixed; and immunostained for α‐tubulin (Cy3; red) and nuclei (DAPI; blue). The panel shows representative images of GFP‐CKAP2L‐expressing, non‐mitotic HEK293 cells in interphase. GFP‐CKAP2L localized to both microtubules and nucleus or to microtubules alone (arrow). (B) Immunostaining of endogenous CKAP2L (Alexa488; green) and α‐tubulin (Cy3; red) shows that some interphase cells exhibit nuclear staining that co‐localizes with DAPI (blue). (C) Western blot showing total cell lysate (Total) or subcellular fractions (Nuclear vs. Cytosolic) of asynchronous HEK293 cells. Lamin A/C was detected to validate the nuclear fraction, and α‐tubulin served largely as a cytosolic marker. Immunoblot for endogenous CKAP2L protein shows that the majority of the protein is found in the nuclear fraction in asynchronously cycling HEK293 cells. Scale bars 10 μm.

**Fig. 5:**
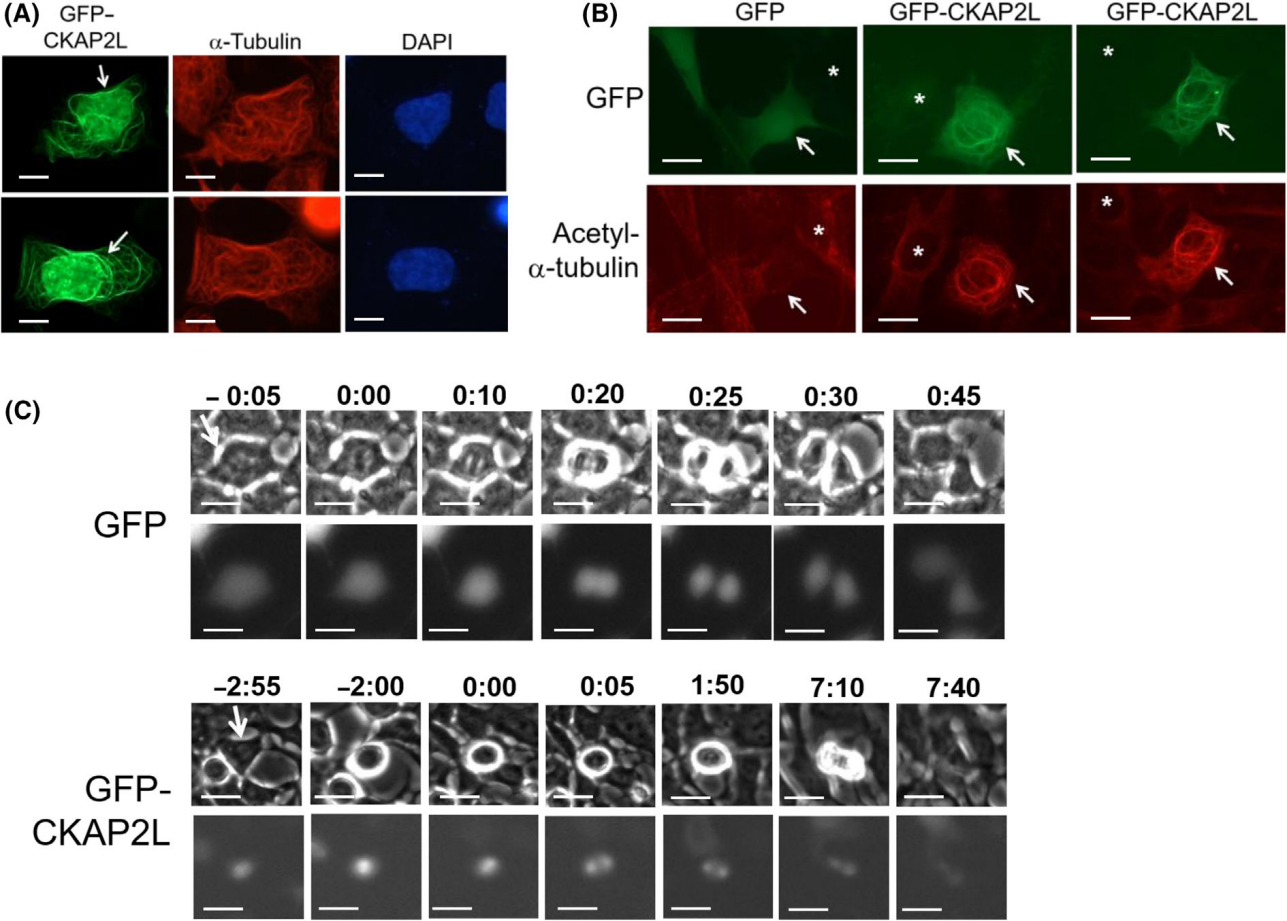
Overexpression of GFP‐CKAP2L increases the stability of microtubules and results in a delay in mitotic progression. (A) HEK293 cells transfected with a GFP‐CKAP2L (green) construct were stained for α‐tubulin (Cy3; red) and nuclei (DAPI; blue). Representative images show ‘microtubule bundling’ (arrows) among GFP‐CKAP2L‐expressing cells. (B) HEK293 cells transfected with a GFP‐CKAP2L (green) construct were immunostained for acetylated α‐tubulin (Cy3; red), an indicator of stable microtubules. Cells transfected with a GFP vector served as a control. GFP or GFP‐CKAP2L‐transfected cells (green) are indicated by arrows. Neighboring, untransfected cells are indicated by asterisks. Strong acetylated α‐tubulin staining (Cy3; red) is only observed in GFP‐CKAP2L‐expressing cells. (C) Time‐lapse microscopy of GFP‐CKAP2L‐expressing HEK293 cells. The upper panels show phase‐contrast images, and the bottom panels show GFP‐CKAP2L fluorescent images. The GFP (control)‐expressing cell (upper panels) undergo mitosis within an hour, while the GFP‐CKAP2L‐expressing cell (bottom panels) takes more than 7 h to complete mitosis. The numbers on top show time lapsed in hours:minutes before and after the initiation of mitosis which was set as 0:00. Scale bars 10 μm.

We have previously reported that both endogenous and ectopically introduced CKAP2 localizes to microtubules and centrosomes in human cells during interphase [7, 24]. Unlike CKAP2, ectopic GFP‐CKAP2L localized not only to microtubules but also to the nucleus in interphase HEK293 cells (Figs 4A and 5A). However, the level of microtubular vs. nuclear GFP‐CKAPL in individual cells varied widely. For instance, some cells exhibited relatively strong nuclear staining, while others exhibited no nuclear staining (Fig. 4A). This prompted us to examine the localization of endogenous CKAP2L during interphase. A fraction of interphase cells exhibited nuclear CKAP2L staining, and cytoplasmic staining was minimal (Fig. 4B). Consistent with this finding, endogenous CKAP2L was enriched in the nuclear fraction and absent in the cytosolic fraction isolated from asynchronous HEK293 cells (Fig. 4C), indicating that endogenous human CKAP2L protein in interphase cells remain largely nuclear prior to entering the mitotic phase.

### CKAP2L overexpression induces microtubule bundling

We sought to confirm a previous finding that CKAP2L can stabilize microtubules [14]. Overexpression of ectopic GFP‐CKAP2L in interphase HEK293 cells resulted in ‘microtubule bundling’, characterized by the formation of thick, stable microtubule bundles (Fig. 5A). It suggested that like CKAP2, CKAP2L has microtubule‐stabilizing properties. In line with this observation, the cells transfected with GFP‐CKAP2L often exhibited microtubules decorated with acetylated α‐tubulin, which is an indicator of stable microtubules [28] (Fig. 5B). Next, we monitored GFP‐CKAP2L‐expressing HEK293 cells in real‐time using time‐lapse microscopy, as they undergo cell cycle and mitosis. The GFP control‐expressing cells often completed the mitotic phase of the cell cycle within 1 h (Fig. 5C; top panels). In contrast, it took longer (e.g., hours) for GFP‐CKAP2L‐expressing cells to complete mitosis, despite most formed bipolar spindles (Fig. 5C; bottom panels). This finding suggests that an excessive level of CKAP2L is detrimental to the normal progression of mitosis, which is consistent with observations made by Yumoto *et al*. [14]. The authors reported that overexpression of Radmis, a murine orthologue of CKAP2L, results in mitotic delay/arrest in both cultured cells and mouse embryos, due to formation of mitotic spindle defects [14]. However, due to technical difficulties, we were only able to observe three representative GFP‐CKAPL‐expressing cells via time‐lapse, and it is difficult to conclude that overexpression of GFP‐CKAP2L results in delays in mitotic progression based on our current data.

## Discussion

One of the novel findings of the present study was that the level of CKAP2L expression changes according to the stages of the cell cycle; its mRNA and protein levels gradually increase after the cells enter the cell cycle and reach their maximum during the G_2_/M phase (see Fig. 1). This finding is also supported by a previous transcriptomic analysis of cell cycle‐dependent changes in gene expression in HeLa cells [25]. This study showed that human *CKAP2L* (previously known as *FLJ40629*) shares the same expression pattern with many well‐known mitotic regulators, including *CKAP2*, *NUSAP1*, *TTK*, *CENPA*, *AURKB*, *TOP2A*, *ANLN*. Thus, CKAP2L belongs to the ‘G_2_/M cluster’ [25]. In addition, an analysis using cyclebase 3.0 (https://cyclebase.org/CyclebaseSearch) [29] showed that *CKAP2L* expression peaks at the G_2_ phase (at 79% of the cell cycle) in humans. Of note, the expression of its paralogue, *CKAP2*, peaks at the G_2_/M phase (at 81% of the cell cycle) according to cyclebase 3.0. Such a pattern of cell cycle‐dependent expression of CKAP2L is in line with a previously proposed role of murine CKAP2L (also known as Radmis) in maintaining mitotic spindle apparatus and chromosome segregation in neural stem/progenitor cells [14].

Accordingly, the tissue‐specific expression pattern of *CKAP2L* and *Ckap2l* in humans and mice may reflect the rate of cell proliferation and cell turnover in the tissue. In our study, relatively high expression of *CKAP2L* and *Ckap2l* was observed in the testis, intestine, and spleen in both humans and mice (see Fig. 2). In line with this finding, CKAP2L protein was readily detectable in various human cancer cell lines tested in our current study (see Fig. 2C). Indeed, recent studies have shown that CKAP2L level is elevated in many types of human cancer, including esophageal squamous cell carcinoma, hepatocellular carcinoma, lung adenocarcinoma, and clear cell renal carcinoma [15, 16, 17, 18]. These observations can be easily attributed to the fact that expression of CKAP2L is upregulated at both transcript and protein levels in cells actively undergoing the cell cycle (see Fig. 1). For instance, the elevated expression of CKAP2L in various types of human cancer may reflect the high rate of cell proliferation which is inherent in malignant tissues.

In cancer literature, an overwhelming number of studies found that various types of human cancer exhibit an elevated level of *CKAP2L* expression, which often correlates with their poor prognosis [15, 16, 18, 19, 20, 21, 30]. Similarly, other proteins that are enriched at the G_2_/M phases of the cell cycle, including CKAP2 and Ki‐67, have been shown to have prognostic values in human cancer [12, 13, 31, 32]. We speculate that the level of the G_2_/M phase markers, such as CKAP2L, reflect the overall cell proliferative rate and, thus, correlates with the “aggressiveness” of the cancer. For this reason, it is currently unclear whether CKAP2L plays a bona fide, oncogenic, or tumor‐promoting role or simply correlates with the proliferative nature of cancer cells. The latter appears to be more plausible since its discovery as a potential cancer biomarker has been linked to other G_2_/M phase regulators, such as *PRC1*, *TOP2A*, *KIF23*, *CCNF*, and *BUB1* [33, 34, 35, 36].

It appears that an excessive level of CKAP2L prevents the proper execution of mitosis. A previous report by Yumoto *et al*. [14] showed that overexpression of CKAP2L not only leads to increased microtubule stability but also defects in mitotic spindle formation and subsequent mitotic arrest. Moreover, the mitotic defects are more pronounced when a “non‐degradable” mutant form of CKAP2L (i.e., KEN box mutant) is introduced [14], suggesting that the CKAP2L protein level needs to be tightly regulated to maintain the fidelity of mitosis. Consistent with this finding, our present study showed that ectopic overexpression of GFP‐CKAP2L in HEK293 cells often resulted in a prolongation of the mitotic phase, even in cells that formed apparent, bipolar spindles (see Fig. 5C). It is likely that an excessive level of CKAP2L and subsequent alterations in spindle microtubule dynamics activate the spindle assembly checkpoint which prevents the metaphase to anaphase transition until proper microtubule‐to‐kinetochore attachments are established [37]. Based on this, we speculate that excessive overexpression of CKAP2L is detrimental not only to normal cells but also to cancer cells. However, one cannot rule out the possibility that CKAP2L may play roles other than regulation of the mitotic spindle and chromosome segregation in cancer cells. One recent study showed that overexpression of *CKAP2L* in esophageal squamous carcinoma cells promotes their growth and migration, and correlates with resistance to flavopiridol, a CDK inhibitor with anticancer effects [17].

Subcellular localization of endogenous human CKAP2L (and GFP‐CKAP2L) during mitosis was largely in agreement with a previous study by Yumoto *et al*. (see Fig. 3). However, the authors reported that, during cytokinesis, CKAP2L localizes to the midbody microtubules in N2a cells. In our study, CKAP2L localized to the centrosomes and was absent from the midbody microtubules (see Fig. 3). The differences in antibodies and cell lines (N2a vs. HEK293) between the two studies might have contributed to this discrepancy. We observed that the localization of GFP‐fusion CKAP2L protein essentially mirrored the antibody staining (see Fig. 3), validating the antibody staining. In addition, we have also observed similar CKAP2L staining patterns in HeLa cells (H. Kwon, unpublished observations). Another important, novel finding of our study was that endogenous CKAP2L protein localizes to the nucleus during interphase, although with weak intensity (see Fig. 4). The interphase cells exhibiting nuclear CKAP2L staining are presumably at the G_2_ phase of the cell cycle, during which its protein level is relatively high. However, this contrasted with GFP‐CKAP2L which showed both microtubular and nuclear localization (see Fig. 4A). We hypothesize that cells that exhibit microtubular localization of GFP‐CKAP2L represent the ones in earlier phases of the cell cycle (e.g., G_1_ or early S‐phase), whereas nuclear GFP‐CKAP2L occurs during S/G_2_ phase. We speculate that the apparent difference in subcellular localization between endogenous CKAP2L and GFP‐CKAP2L is caused by the difference in the cell cycle‐dependent expression pattern of endogenous vs. ectopic CKAP2L expression. The level endogenous CKAP2L protein is regulated according to the cell cycle (that it is relatively low or absent during G_1_; starts to rise during S; peaks at G_2_/M phases). In contrast, GFP‐CKAP2L expression is driven by a constitutively active and strong CMV promoter, and as a result, asynchronous cells at different phases of the cell cycle (e.g., including cells at G_0_/G_1_ in which endogenous CKAP2L is hardly detected) express detectable levels of the GFP‐fusion protein. This idea is supported by Fig. S5. It has been previously reported that strong nuclear PCNA staining indicates cells in the S‐phase [38]. HEK293 cells showing mostly microtubular and cytoplasmic localization of GFP‐CKAP2L showed relatively weak PCNA staining, whereas the ones showing strong nuclear PCNA staining had predominantly nuclear GFP‐CKAP2L (see Fig. S5A). Additionally, GFP‐CKAP2L in recently divided two daughter cells (and thus are likely in the G_0_/G_1_ phase of the cell cycle) were mainly localized to the cytoplasm and microtubules, instead of the nucleus (see Fig. S5B).

CKAP2L and its only paralogue, CKAP2, seem to share biochemical and functional attributes, despite that they only share 16% amino acid identity [14]. CKAP2L harbors ‘Cytoskeleton‐associated protein 2, C‐terminal’ domain (InterPro IPR029197) at its C‐terminus (i.e., 423–734 aa) which exhibits greater homology with CKAP2. For example, the ^682^KFITPVRRSSRI^693^ motif (UniProt Q8IYA6‐1) which is present in its C‐terminus is conserved in multiple species and conserved in CKAP2 (UniProt Q8WWK9‐5), as in ^619^KFLTPVRRSRRL^630^. We have previously reported that Thr622 and Ser627 (of ^619^KFL**T**PVRR**S**RRL^630^; residues in bold) of human CKAP2 are phosphorylated during mitosis by Cdk1/cyclin B1 and Aurora B kinase, respectively [9, 39]. Thus, it is likely that the corresponding residues in CKAP2L, i.e., Thr685 and Ser690 (of ^682^KFI**T**PVRR**S**SRI^693^; residues in bold), are phosphorylated by these mitotic kinases as well. Global phospho‐proteome analyses have shown that Thr685 is indeed phosphorylated during mitosis [40, 41]. However, it would require additional studies to identify these residues as bona fide phosphorylation sites *in vivo*.

To our knowledge, the functional importance of CKAP2L phosphorylation, however, has never been explored. CKAP2L and CKAP2 proteins may share the same mechanism of protein degradation during mitotic exit. We and others have shown that CKAP2 is subject to degradation by the anaphase promoting complex/cyclosome (APC/C)‐Cdh1 complex via its KEN box motif [7, 10]. Yumoto *et al*. reported that an evolutionarily conserved KEN box motif is present in CKAP2L (i.e., ^186^KEN^187^; UniProt Q8IYA6‐1). When ^186^KEN^187^ box was mutated to ^186^AAA^187^ and introduced into a mouse embryo in utero via electroporation, a mutant KEN box‐CKAP2L shows enhanced protein stability *in vivo* and caused a greater frequency of mitotic defects in neural progenitor cells [14], suggesting that degradation of CKAP2L during mitotic exit by APC/C‐Cdh1 complex is of functional importance. However, the authors did not provide experimental evidence to demonstrate that CKAP2L is a direct substrate of APC/C‐Cdh1. Our analysis shows that CKAP2L has at least two, conserved D‐box's (R‐x‐x‐L), a destruction motif recognized by APC/C‐Cdc20 [42]. In addition, the kinetics of degradation of CKAP2L during mitotic exit appear to coincide with those of cyclin B1 (see Fig. 1D) which is degraded by the APC/C‐Cdc20 complex prior to the onset of anaphase [43, 44]. Thus, it remains to be seen whether CKAP2L protein stability is regulated solely by APC/C‐Cdh1 during mitotic exit.

Mutations in CKAP2L have been shown to cause a rare genetic disease called Filippi syndrome [5], which highlights the important role of CKAP2L in neurogenesis. Accordingly, shRNA‐induced knockdown of murine *Ckap2l* (Radmis) induces formation of multipolar mitotic spindles and defects in chromosome segregation in cultured NIH3T3/13C7 cells which include long chromosome bridge formations between two separating daughter nuclei and misalignment of metaphase chromosomes in neural progenitor cells in the developing mouse brain [14]. A separate group of researchers reported that siRNA‐mediated depletion of CKAP2L in esophageal squamous cell carcinoma cells in culture increases the percentage of cells at the G_2_/M phase [17]. Others reported that depletion of CKAP2L suppressed proliferation, migration, and invasion of lung adenocarcinoma cells, including H460, A549, and H1299 [15, 30]. Notably, these studies in cancer cells, however, did not specifically investigate the defects during mitosis in CKAP2L‐depleted cells. Taken together, these previous studies suggest that CKAP2L is required for the growth of both normal and malignant cell types, presumably via maintaining the fidelity of mitotic spindle apparatus and chromosome segregation. Similarly, we have previously reported that its paralogue, CKAP2, is essential for proper chromosome segregation and maintenance of genomic stability [8].

In our present study, we showed that CKAP2L shares similar characteristics and functional attributes with its paralogue, CKAP2. However, there were notable differences between CKAP2L and CKAP2, particularly in subcellular localization. For instance, the endogenous CKAP2L is mainly in the nucleus (see Fig. 4) during interphase whereas CKAP2 localizes with the microtubules [6, 10, 24]. Moreover, during late mitosis/cytokinesis when CKAP2L protein level declines, the remaining CKAP2L is localized to the nascent daughter cell centrosomes (see Fig. 3A). In contrast, starting at some point during anaphase B, CKAP2 translocates to the chromatin region and is no longer associated with spindle or midzone microtubules by telophase [7, 45]. CKAP2 is later found in the nascent daughter cell nuclei and midbody microtubules during late mitosis/cytokinesis [7, 45]. Although the molecular basis of these differences is currently unknown, it should be noted that the region of relatively high homology between two proteins is limited to approximately 310 amino acids at their C‐termini. It requires additional studies to decipher the functional similarities and differences between CKAP2L and CKAP2 with respect to their roles in regulation of microtubule dynamics and mitosis.

## Conclusions

Human CKAP2L is a mitotic spindle‐associated protein and the only known paralog of another spindle‐associated protein, CKAP2. In normal human fibroblasts, the transcript and protein levels of CKAP2L show a cell cycle‐dependent expression pattern and peak at G_2_/M phases of the cell cycle. Among organs and tissues, testis, intestine, and spleen show relatively high levels of the CKAP2L transcript. It is also expressed and detectable in multiple human cancer cell lines with different tissue origins. Human CKAP2L protein shows a cell cycle‐dependent subcellular localization pattern. During interphase, it is mainly located within the nucleus. Upon entry into mitosis, it localizes to the spindle poles and microtubules. CKAP2L exhibits microtubule‐stabilizing properties.

## Conflict of interest

The authors declare no conflict of interest.

### Peer review

The peer review history for this article is available at https://www.webofscience.com/api/gateway/wos/peer‐review/10.1002/2211‐5463.13864.

## Author contributions

HK contributed to validation, formal analysis, investigation, visualization, data curation, writing‐reviewing, and editing. JYJ contributed to data curation, writing‐original draft preparation, writing‐ reviewing and editing. KUH contributed to conceptualization, methodology, data curation, supervision, visualization, project administration, writing‐original draft preparation, writing‐reviewing and editing.

## Supporting information


**Fig. S1.** Characterization of affinity‐purified rabbit polyclonal anti‐human CKAP2L antibody.
**Fig. S2.** Characterization of affinity‐purified rabbit polyclonal anti‐human CKAP2L antibody.
**Fig. S3.** Flow cytometry data presented in Fig. 1B showing both axes.
**Fig. S4.** Changes in subcellular localization of CKAP2L between late anaphase and cytokinesis.
**Fig. S5.** Evidence for cell cycle‐dependent subcellular localization of CKAP2L.

## Data Availability

The data that support the findings of this study are available from the corresponding author (kyung.hong@wne.edu) upon reasonable request.
