# Supplementary material for: Human CKAP2L shows a cell cycle‐dependent expression pattern and exhibits microtubule‐stabilizing properties
Source: FEBS Open Bio. 2024 Jul 28;14(9):1526–39. doi: 10.1002/2211-5463.13864 (PMC11492392; doi:10.1002/2211-5463.13864)
Supplement: Supplementary file 1 — Fig. S1. Characterization of affinity‐purified rabbit polyclonal anti‐human CKAP2L antibody. Fig. S2. Characterization of affinity‐purified rabbit polyclonal anti‐human CKAP2L antibody. Fig. S3. Flow cytometry data presented in Fig. 1B showing both axes. Fig. S4. Changes in subcellular localization of CKAP2L between late anaphase and cytokinesis. Fig. S5. Evidence for cell cycle‐dependent subcellular localization of CKAP2L. [file FEB4-14-1526-s001.pdf]

Supplemental Materials

Supplemental Figure S1.

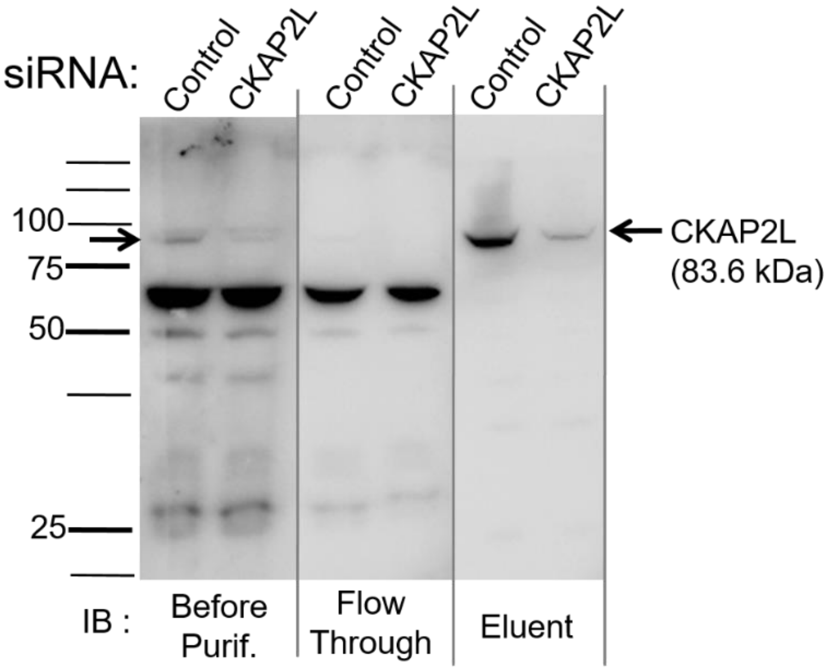

Figure S1. Characterization of affinity-purified rabbit polyclonal anti-human CKAP2L antibody. Rabbits were immunized with 1-455 aa of human CKAP2L and the resulting serum was affinity-purified for CKAP2L-specific polyclonal antibodies. Whole cell lysate of HEK293 cells transfected with control siRNA (Control) or human *CKAP2L*-specific siRNA (CKAP2L) was separated by SDS-PAGE and immunoblotted with the indicated fraction: 1) crude serum (Before Purif.); 2) flow through from affinity chromatography column (Flow Through); 3) affinity-purified CKAP2L antibody (Eluent). In affinity-purified, rabbit polyclonal anti-human CKAP2L antibody (Eluent) detects a single, major band between 75 and 100 kDa molecular markers (see arrow). The band intensity is reduced in the CKAP2L siRNA-transfected sample, which validates the specificity of the antibody. The target sequence for human *CKAP2L* siRNA was the following: 5'-ACACCAAGCCTTATCTAAA-3'. The control siRNA had a scrambled sequence with no known targets.

Supplemental Figure S2.

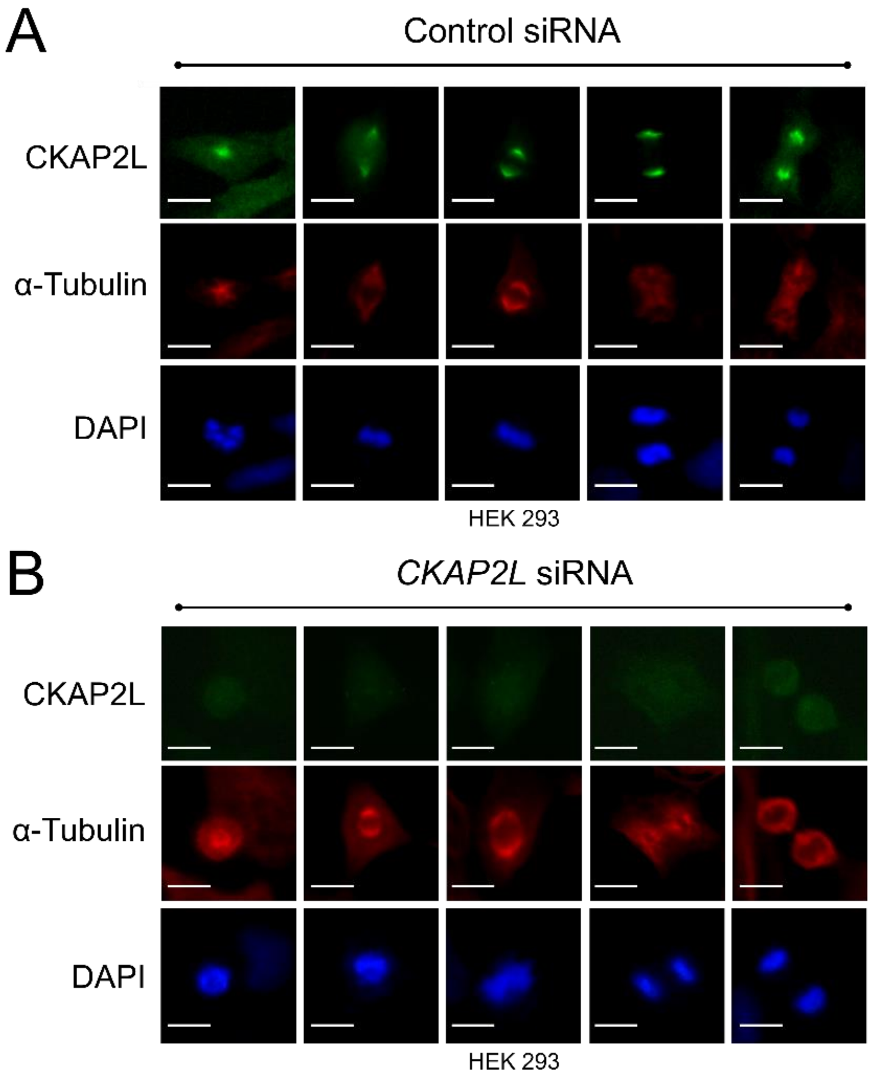

Figure S2. Characterization of affinity-purified rabbit polyclonal anti-human CKAP2L antibody. HEK293 cells were transfected with control siRNA or human *CKAP2L*-specific siRNA (CKAP2L). After 2 days of transfection, the cells were fixed and co-immunostained with a rabbit polyclonal anti-human CKAP2L antibody (Alexa488; green) and a monoclonal antibody against α-tubulin (Cy3; red). DAPI was used to stain nuclei (blue). Panels show representative images of cells at different phases of mitosis. The CKAP2L staining at the mitotic apparatus in control siRNA-transfected cells (see Panel A) was gone in CKAP2L siRNA-transfected cells, demonstrating the specificity of the CKAP2L antibody staining. The target sequence for human *CKAP2L* siRNA was the following: 5'-ACACCAAGCCTTATCTAAA-3'. The control siRNA had a scrambled sequence with no known targets. Scale bars 10 μm.

Supplemental Figure S3.

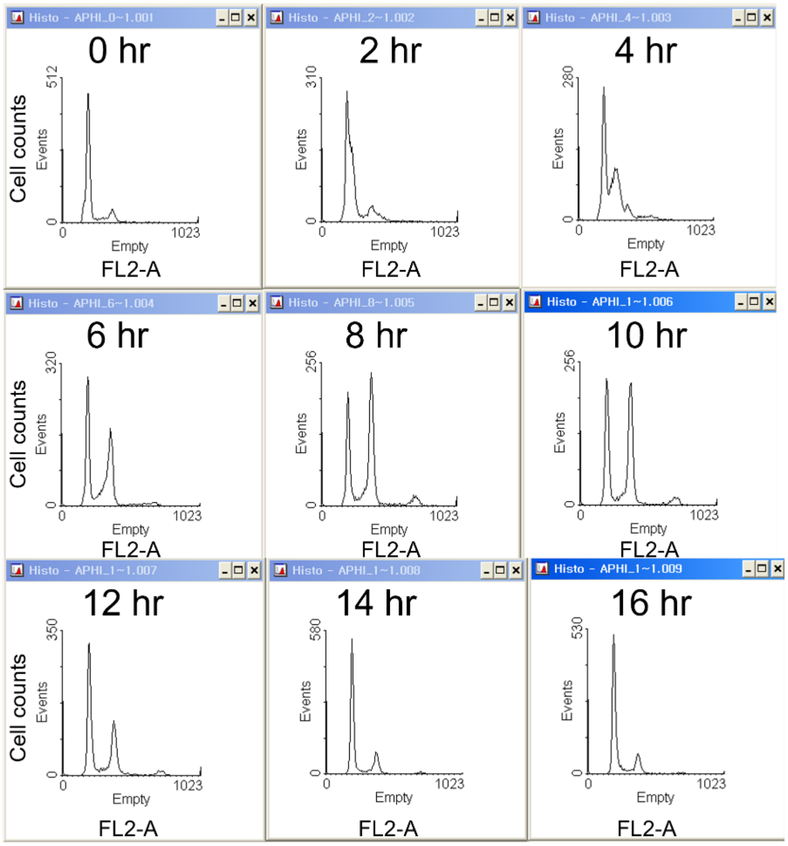

Figure S3. Flow cytometry data presented in Figure 1B showing both axes. Normal human foreskin fibroblasts were arrested at the G_1-S boundary using aphidicolin; released; and analyzed at the indicated time points. On the y-axis, Events are equivalents to the number of cells (Cell counts). On the x-axis, FL2-A is a measure of relative propidium iodide intensity.

Supplemental Figure S4.

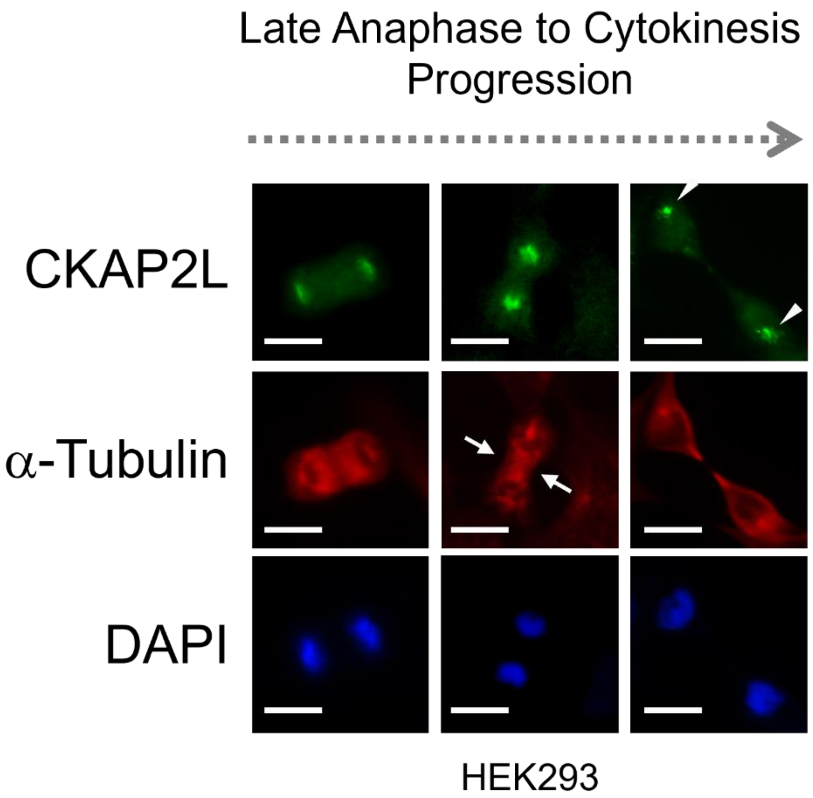

Figure S4. Changes in subcellular localization of CKAP2L between late anaphase and cytokinesis. HEK293 cells were fixed and co-immunostained with a rabbit polyclonal anti-human CKAP2L antibody (Alexa488; green) and a monoclonal antibody against α-tubulin (Cy3; red). DAPI was used to stain nuclei (blue). Panels show representative images of cells at different phases of mitosis between late anaphase and cytokinesis. Towards the completion of cytokinesis, CKAP2L staining becomes restricted to the nascent centrosomes (panels on the right; white arrows). White arrows indicate the cleavage furrow formation. Scale bars 10 μm.

Supplemental Figure S5.

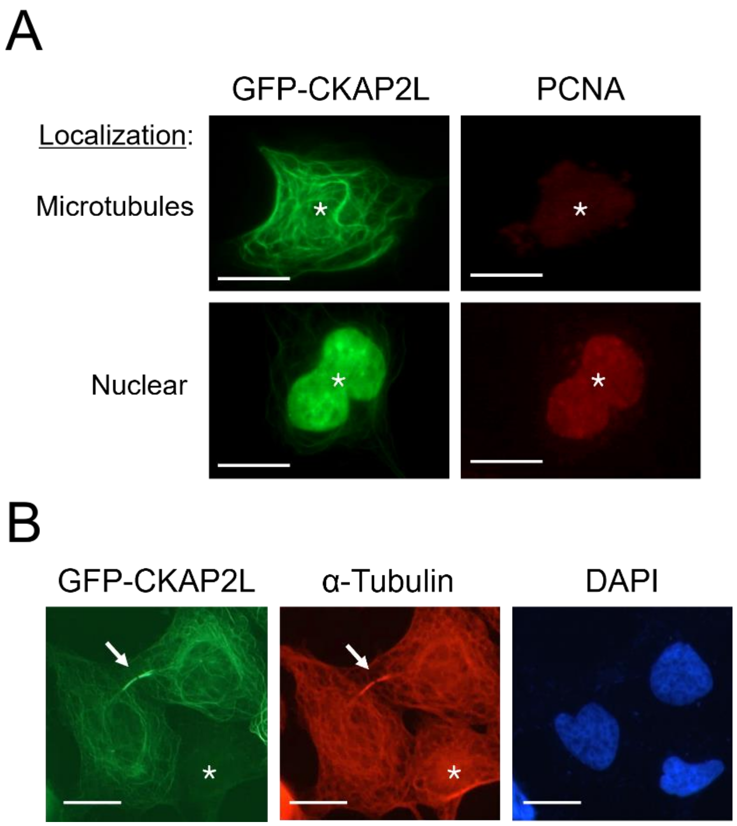

Figure S5. Evidence for cell cycle-dependent subcellular localization of CKAP2L. HEK293 cells transfected with a GFP-CKAP2L (green) construct were stained for PCNA (Panel A; red) α-tubulin (Panel B; red) and nuclei (DAPI; blue). It has been previously reported that strong nuclear PCNA staining indicates cells in the S-phase [38]. A, the cells showing mostly microtubular and cytoplasmic localization of GFP-CKAP2L (green) showed relatively weak PCNA staining, whereas the ones showing strong nuclear PCNA staining (red) showed predominantly nuclear GFP-CKAP2L. Asterisks mark the locations of the nuclei. B, a post-mitotic bridge between two daughter cells suggests that the cells have undergone a recent cytokinesis and are likely in the G_0/G_1 phase of the cell cycle. GFP-CKAP2L (green) in these cells were mainly localized to the cytoplasm and microtubules (α-Tubulin), instead of the nucleus (DAPI). Asterisk indicates a neighboring, non-transfected cell. Scale bars 10 μm.
